# ATP11B Modulates Microglial Lipid Metabolism and Alleviates Alzheimer's Disease Pathology

**DOI:** 10.1002/mco2.70139

**Published:** 2025-03-22

**Authors:** Yuchen Zhang, Shibo Zhang, Xuyu Zhao, Peiru Wu, Yiwei Ying, Lingling Wu, Junyi Zhuang, Zixin Chen, Yufan Chao, Xin Dong, Robert Chunhua Zhao, Jiao Wang

**Affiliations:** ^1^ School of Life Sciences Shanghai University Shanghai China; ^2^ School of Medicine Shanghai University Shanghai China; ^3^ Institute of Basic Medical Sciences Chinese Academy of Medical Sciences, School of Basic Medicine Peking Union Medical College Beijing China; ^4^ Centre of Excellence in Tissue Engineering Chinese Academy of Medical Sciences Beijing China; ^5^ Beijing Key Laboratory of New Drug Development and Clinical Trial of Stem Cell Therapy (BZ0381) Beijing China

**Keywords:** Alzheimer's disease, ATP11B, lipid droplets, lipid metabolism, microglia

## Abstract

Abnormal lipid metabolism in microglia leads to the formation of pathological lipid droplets (LDs), a phenomenon also observed in neurodegenerative diseases such as Alzheimer's disease (AD). The abnormal accumulation of LDs disrupts normal cellular function and exacerbates the pathological process of AD. ATP11B is a P4‐ATPase and the expression of *Atp11b* changes in the brain of patients with AD and diseases of lipid metabolism. The present study aimed to explore the regulatory role of ATP11B in microglial lipid metabolism and assess the potential of ATP11B as a therapeutic target for AD. *Atp11b* deficiency caused excessive fatty acid uptake and activated the PPAR signaling pathway, resulting in abnormal synthesis of neutral lipids and mitochondrial energy metabolism in microglia. Further results showed that *Atp11b* deficiency led to the accumulation of pathological LDs in microglia and AD mice. Conversely, overexpression of *Atp11b* alleviated exploratory behavior impairment, learning and memory impairment, LD accumulation, beta‐amyloid (Aβ) deposition, and inflammatory response in the brain of AD mice. These findings provide important clues for a better understanding of the pathogenesis of AD and for developing novel therapeutic strategies.

## Graphical abstract

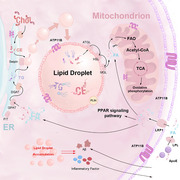

## 1 Introduction

Lipid metabolism plays a crucial role in energy metabolism, cellular functions, and overall organism health. The endoplasmic reticulum (ER) is the primary organelle for neutral lipid synthesis [1]. The assembly of neutral lipids into lipid droplets (LDs) leads to a reduction in fatty acids (FAs) in microglia while the FAs released through LD lipolysis or lipophagy can be utilized by mitochondria [2]. LDs are essential cellular lipid storage organelles that regulate the storage and regulation of lipid metabolism in microglia [2]. LDs consist of an outer monolayer of phospholipids and inner neutral lipids, mainly triglycerides (TGs) or cholesterol esters (CEs) [1]. The abnormal formation of LDs in microglia is caused by several factors including disruptions in the lipid metabolism pathway, increased levels of reactive oxygen species (ROS), mitochondrial dysfunction, and intensified inflammatory response [3, 4]. LD accumulation in microglia further leads to neuroinflammation and neurodegeneration in the central nervous system (CNS), contributing to the development of neurodegenerative diseases [5, 6, 7, 8, 9].

Microglia, a vital component of the CNS, play a crucial role in maintaining homeostasis. A recent study by Marschallinger et al. [10] identified a new subtype of microglia in the hippocampus, known as “lipid droplet accumulating microglia (LDAM).” LDAM exhibits a distinctive transcriptional profile and gradually accumulates LDs, produces ROS, and releases proinflammatory factors as individuals age [10]. LDAM has been observed in the brains of Alzheimer's disease (AD) patients, indicating altered lipid metabolism in these cells [11]. This discovery suggests that dysregulated lipid metabolism in microglia may contribute to the pathogenesis of AD, highlighting the importance of further research into the mechanisms underlying LD accumulation in AD.

The prevalence of LDs in the development and progression of AD has been the subject of numerous studies over recent decades. Studies have shown that in the CNS of AD patients, the number of LDs increases significantly and LDs are mainly distributed in neurons and glial cells [12, 13]. The relationship between AD and LDs in the brain was first identified in 1907 when LDs were noted as adipose saccules in glial cells alongside extracellular plaques formed by beta‐amyloid (Aβ) and neurofibrillary tangles formed by phosphorylated‐tau (p‐tau) in neurons [14]. Moreover, LD accumulation has been found to precede the formation of Aβ plaques and neurofibrillary tangles formation in mouse models of AD, but It remains controversial whether the Ab and tau protein deposition are the main reasons or the results of the AD neurodegenerative processes [15, 16]. However, this suggests that LDs may serve as early indicators and promoters of neurodegeneration [17, 18]. On the one hand, LDs directly affect the pathological process of AD by promoting the accumulation of Aβ and p‐tau [19, 20, 21]. Reducing the level of TG to decrease LD numbers could alleviate the accumulation of p‐tau [22]. On the other hand, LD accumulation contributes to abnormal mitochondrial function, heightened oxidative stress, and an intensified inflammatory response, indirectly exacerbating the pathological process of AD [4, 23, 24, 25, 26]. These findings suggest that altering the molecular mechanisms of lipid metabolism and LD formation may be a promising therapeutic approach to AD.

ATP11B is a type of P4‐ATPases that flips phospholipids across membranes. Initial phase of this study has shown that the level of *Atp11b* in the brain of AD patients decreased significantly (GSE160936, GSE175814, and GSE188545). Additionally, abnormal expression of *Atp11b* has been observed in samples from individuals with disorders of lipid metabolism or lipid accumulation in the CNS (GSE190451, GSE217719, GSE244091). These results suggest a potential link between ATP11B and abnormal lipid metabolism in human disease. ATP11B is involved in regulating the transport and distribution of lipid molecules [27, 28]. It is reasonable to suggest that the deficiency in *Atp11b* may disrupt the transport of lipid molecules between cell membranes and membrane organelles, thereby affecting processes such as lipid uptake, lipid synthesis in the ER, lipid degradation, and fatty acid oxidation (FAO) in mitochondria.

The present study aimed to investigate the impact of *Atp11b* deficiency on the lipid metabolism disorder of microglia and its potential application in the treatment of AD. By examining the association between lipid metabolism, LDs, and AD, we endeavor to further our understanding of the pathogenesis of AD and uncover new insights for therapeutic strategies. This research will contribute to elucidating the molecular mechanism by which ATP11B inhibits LD formation and exploring the potential role of ATP11B in the treatment of AD.

## 2 Results

### 2.1 *Atp11b* Deficiency Alters the Lipid Composition of Microglia as Revealed by Lipidomics

**FIGURE 1:**
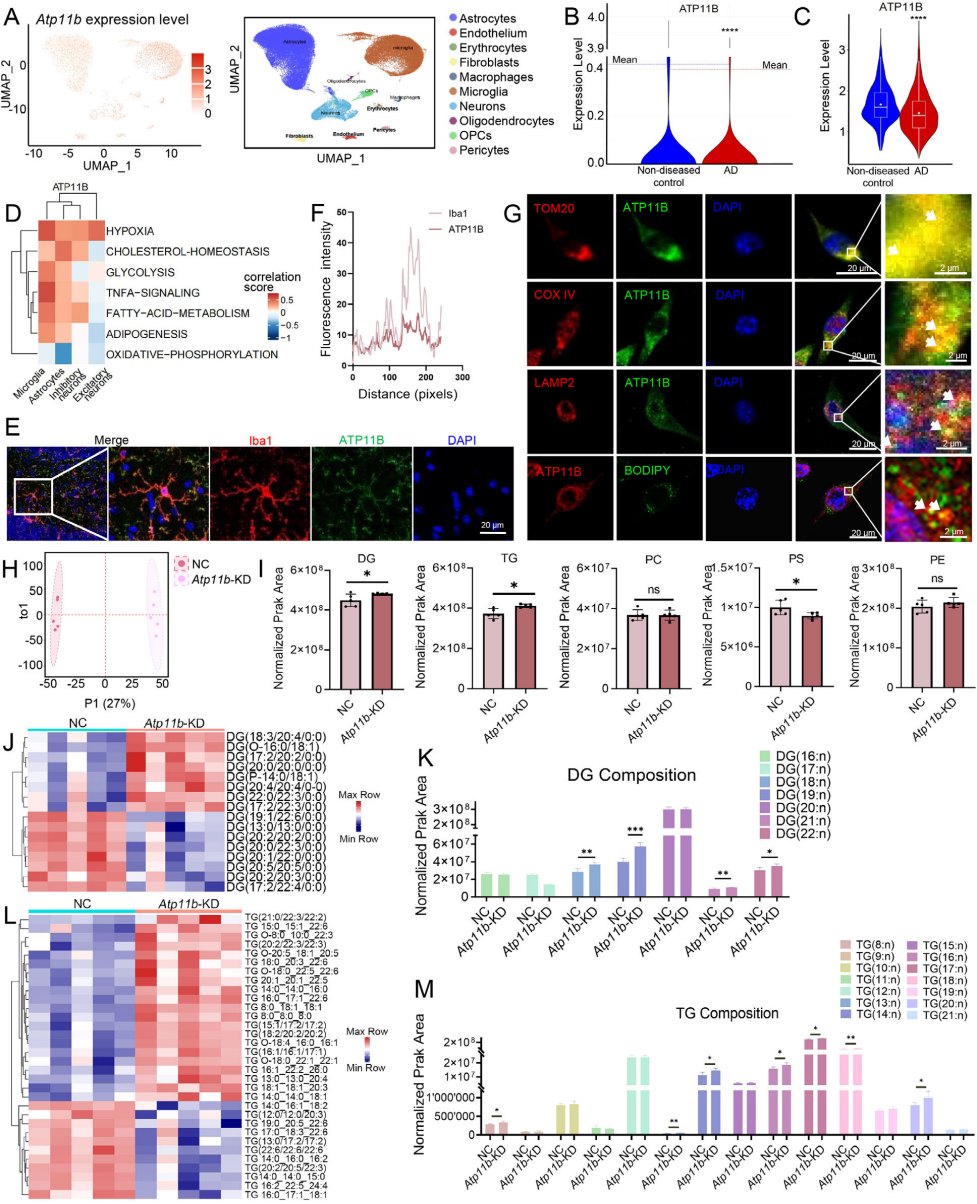
*Atp11b* deficiency alters the lipid composition of microglia as revealed by lipidomics. (A) Umap showed the distribution of ATP11B in various cells in the brain of AD patients from the GSE160936 dataset. (B) The expression level of *Atp11b* from the GSE160936 dataset. Two‐tailed Student's *t*‐test (*n* = 5 of two experiments). (C) The expression level of *Atp11b* from the GSE175814 and GSE188545 datasets. Two‐tailed Student's *t*‐test (*n* = 5 of two experiments). (D) Pearson correlation analysis of ATP11B and KEGG enriched pathways. (E) Representative images of the hippocampus of C57 mouse immunostained for Iba1 (microglia), ATP11B, and DAPI. (F) Quantification of fluorescence intensity of Iba1 and ATP11B with distance change in panel (E). (G) Representative images of immunofluorescence colocalization of TOM20 (mitochondria), COXIV (mitochondria), LAMP2 (lysosomes), and BODIPY (LDs) with ATP11B. (H) OPLS‐DA plot of the profile of *Atp11b*‐KD BV2 cells relative to NC. (I) Lipidome profiling of DG, TG, PS, PC, and PE content. Two‐tailed Student's *t*‐test (*n* = 5 of two experiments). (J and K) Heat map (J) and composition (K) of DGs that were found dysregulated between NC and *Atp11b*‐KD BV2 cells. Two‐tailed Student's *t*‐test (*n* = 5 of two experiments). (L and M) Heat map (L) and composition (M) of TGs that were found dysregulated between NC and *Atp11b*‐KD BV2 cells. Two‐tailed Student's *t*‐test (*n* = 5 of two experiments).

Based on data in the Gene Expression Omnibus, ATP11B was found to be distributed in various cells in the brain of AD patients (Figure 1A). Additionally, there was a significant decrease in the expression of *Atp11b* of the brain tissue of AD patients (GSE160936) (Figure 1B) [29]. Single‐cell RNA sequencing data from AD patient brains showed a significant decrease of ATP11B expression in the microglia of AD patients (GSE175814, GSE188545) (Figure 1C). This indicates that ATP11B deficiency occurs in AD patient microglia. Moreover, Pearson correlation analysis of ATP11B and KEGG enriched pathways revealed a strong correlation between ATP11B and cholesterol‐homeostasis, glycolysis, FA metabolism, and adipogenesis in the microglia of AD patients (GSE188545) (Figure 1D). This suggests that ATP11B is involved in the regulation of microglial lipid metabolism in AD patients. We also collected the data from human samples of diseases with abnormal lipid metabolism in the CNS and results showed the level of *Atp11b* expression was abnormal (GSE190451, GSE217719, GSE244091) (Figure S1A) [30, 31]. Additionally, the expression levels of *Atp11b* in iMGLs and SV40 cells with ApoE ε4 allele were decreased (GSE203019, GSE163857, GSE193513) (Figure S1B) [26, 32, 33]. These results further indicated the potential relationship between ATP11B and diseases with abnormal lipid metabolism in microglia.

We conducted a study to determine the localization of ATP11B in the CNS. The results of our immunofluorescence analysis revealed that ATP11B is a membrane protein highly expressed in mouse brain microglia (Figure 1E,F). Our observations also demonstrated the colocalization of ATP11B with intracellular organelles associated with lipid metabolism, including mitochondria, lysosomes, and LDs (Figure 1G). Additionally, ATP11B was also present on the ER membrane [27, 34]. These findings strongly suggest that ATP11B is situated on the membranes of these lipid metabolism‐related organelles. In summary, we propose that ATP11B plays a role in regulating the transport and storage of lipids between these membrane‐bound organelles, thereby influencing lipid metabolism.

We utilized siRNA to silence *Atp11b* (hereinafter referred to as *Atp11b*‐knock down (*Atp11b*‐KD)) in BV2 cells to better understanding the function of ATP11B. To preliminarily assess the impact of ATP11B on lipid metabolism in microglia, we conducted lipidomics profiling using ultrahigh‐performance liquid chromatography coupled with Q‐Exactive MS (UPLC‐QE MS) on negative control (hereinafter referred to as NC) and *Atp11b*‐KD BV2 cells. We initially enhanced lipid metabolism in BV2 cells by exposing them to oleic acid (OA). OA treatment effectively led to the oversaturation of FAs within the cells, reflecting significant alterations in lipid metabolism associated with LD formation [26]. The difference between NC and *Atp11b*‐KD BV2 cells was compared by orthogonal partial least squares discriminant analysis (OPLS‐DA) (Figure 1H) and principal component analysis (Figure S2A). As expected, *Atp11b*‐KD led to a distinctive lipid profile. In addition, *Atp11b*‐KD caused differences in the content of 1125 lipids, of which 561 were upregulated and 564 were downregulated (Figure S2B). We compared the overall expression levels of major lipids, including diacylglycerol (DG), TG, phosphatidylserine (PS), phosphatidylcholine (PC), and phosphatidylethanolamine (PE) in NC and *Atp11b*‐KD BV2 cells. The results showed that there were significant differences in the content of DG, TG, and PS, among which DG and TG were significantly upregulated, while PS was significantly downregulated in *Atp11b*‐KD BV2 cells (Figure 1I). DG and TG analysis determined that ATP11B affected the accumulation and metabolism of neutral lipids in BV2 cells.

The impact of ATP11B on neutral lipids was further investigated by conducting a detailed analysis of DG and TG. Specifically, the differential metabolites of DGs in *Atp11b*‐KD BV2 cells are shown in the figure (Figure 1J). *Atp11b*‐KD significantly increased the DGs of FA carbon chain numbers 18, 19, 21, and 22 in BV2 cells (Figure 1K). Meanwhile, the differential metabolites of TGs in *Atp11b*‐KD BV2 cells are shown in the figure (Figure 1L). *Atp11b*‐KD significantly increased the TGs of FA carbon chain numbers 8, 13, 14, 16, 17, 18, and 20 in BV2 cells (Figure 1M). Furthermore, an analysis of phospholipids revealed significant differences in PS metabolites with carbon chain lengths of 12 and 20, while no significant variations were noted between PC and PE metabolites (Figure S2C–E). In summary, the lipidomics results suggest that *Atp11b* deficiency primarily alters the composition of neutral lipids in BV2 cells, implying the accumulation of neutral lipids in BV2 cells.

### 2.2 *Atp11b*‐Deficient Brain Microglia Display Increased Lipid Uptake

**FIGURE 2:**
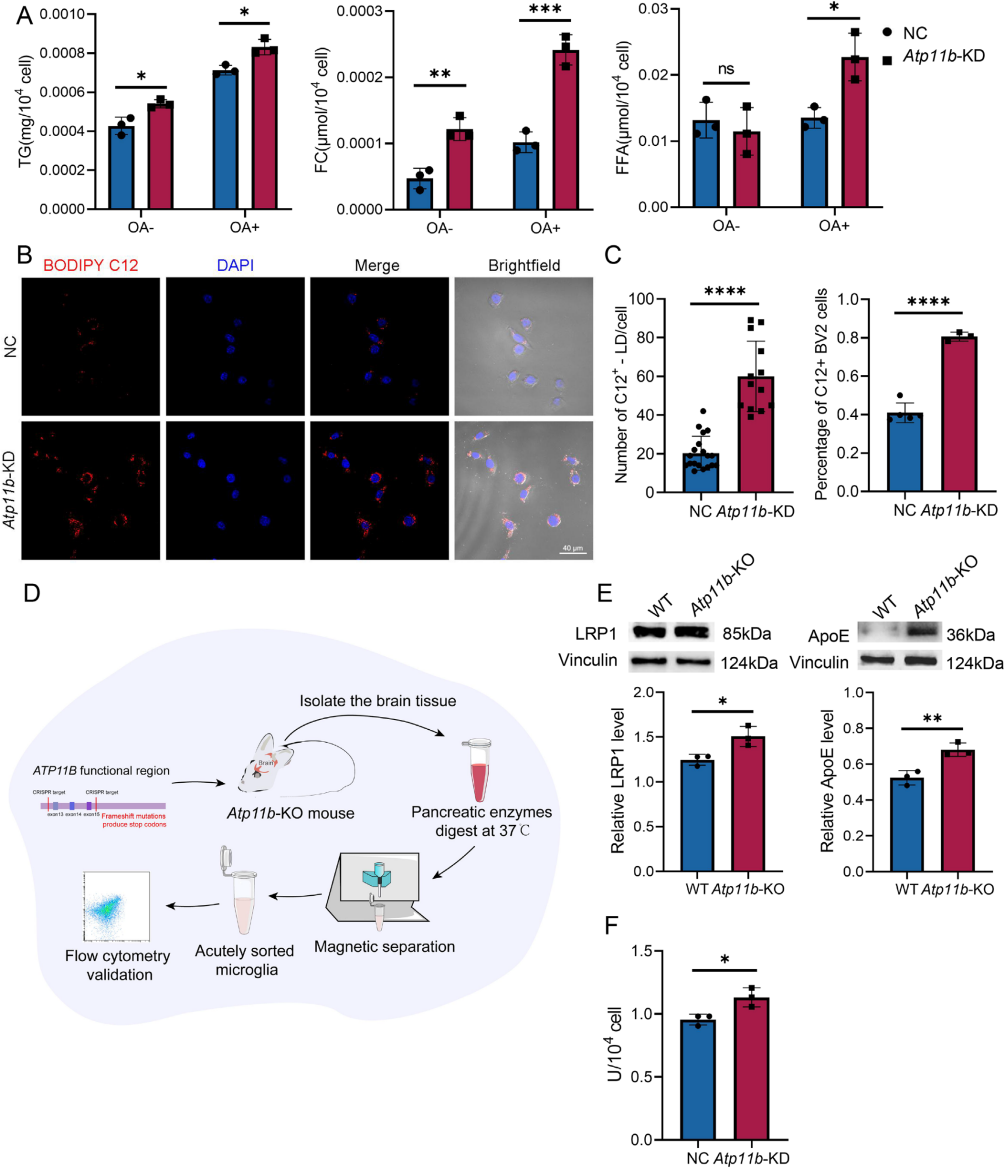
*Atp11b*‐deficient brain microglia display increased lipid uptake. (A) Quantification of TG, FC and, FFA content in NC and *Atp11b*‐KD BV2 cells treated with OA (1 mM) for 12 h. One‐way ANOVA (*n* = 3). (B) Representative images of NC and *Atp11b*‐KD BV2 cells immunostained for BODIPY C12 and DAPI. (C) Quantification of the percentage of C12+ BV2 cells and the number of C12+ LD per cell. Two‐tailed Student's *t*‐test (*n* = 3 of two experiments). (D) Construction diagram of acutely isolated microglia in the brain of *Atp11b*‐KO mice. (E) Western blot images and quantification of LRP1 and ApoE level in microglia acutely sorted from 6 M WT and *Atp11b*‐KO mice. Two‐tailed Student's *t*‐test (*n* = 3 of two experiments). (F) Quantification of LPL enzyme activity in NC and *Atp11b*‐KD BV2 cells. Two‐tailed Student's *t*‐test (*n* = 3 of two experiments).

The lipidomics analysis revealed that *Atp11b*‐KD led to an increase in neutral lipids in BV2 cells. This was further supported by the measurement of TG content in BV2 cells with or without OA treatment (Figure 2A). In addition, *Atp11b*‐KD increased the content of free cholesterol (FC) in BV2 cells, but the content of free fatty acid (FFA) did not change (Figure 2A). CE and TG are the main neutral lipids in BV2 cells and FC is stored in LDs as CEs [35]. We postulated that *Atp11b* deficiency may promote lipid uptake, leading to the observed increase in neutral lipids.

We introduced BODIPY‐C12 to *Atp11b*‐KD BV2 cell culture medium to explore their ability to uptake FFAs. Our findings revealed that *Atp11b*‐KD significantly increased FA uptake by BV2 cells (Figure 2B,C). Low‐density lipoprotein receptor‐associated protein 1 (LRP1) is a multifunctional transmembrane receptor with endocytosis and signal transduction properties [36]. Extracellular lipoproteins containing ApoE transport lipids to BV2 cells and bind to LRP1. Then, the lipids carried by ApoE are hydrolyzed by the cell membrane lipoprotein lipase (LPL) and taken up by the cell [37, 38]. To verify the function of ATP11B in vivo, we acutely isolated microglia from laboratory‐constructed *Atp11b* ^−/−^ mice (hereinafter referred to as *Atp11b*‐knock out (*Atp11b*‐KO)) [39] (Figure 2D). We also assayed the gene expression level of *Atp11b* in acutely isolated microglia (Figure S3A). Western blot results showed that LRP1 and ApoE levels were higher in microglia acutely isolated from *Atp11b*‐KO mice than wild type (WT) mice (Figure 2E). Also, the activity of LPL was enhanced in *Atp11b*‐KD BV2 cells (Figure 2F). These results suggest that *Atp11b* deficiency promoted microglial uptake.

### 2.3 *Atp11b* Silencing Activates the PPAR Signaling Pathway in Microglia

**FIGURE 3:**
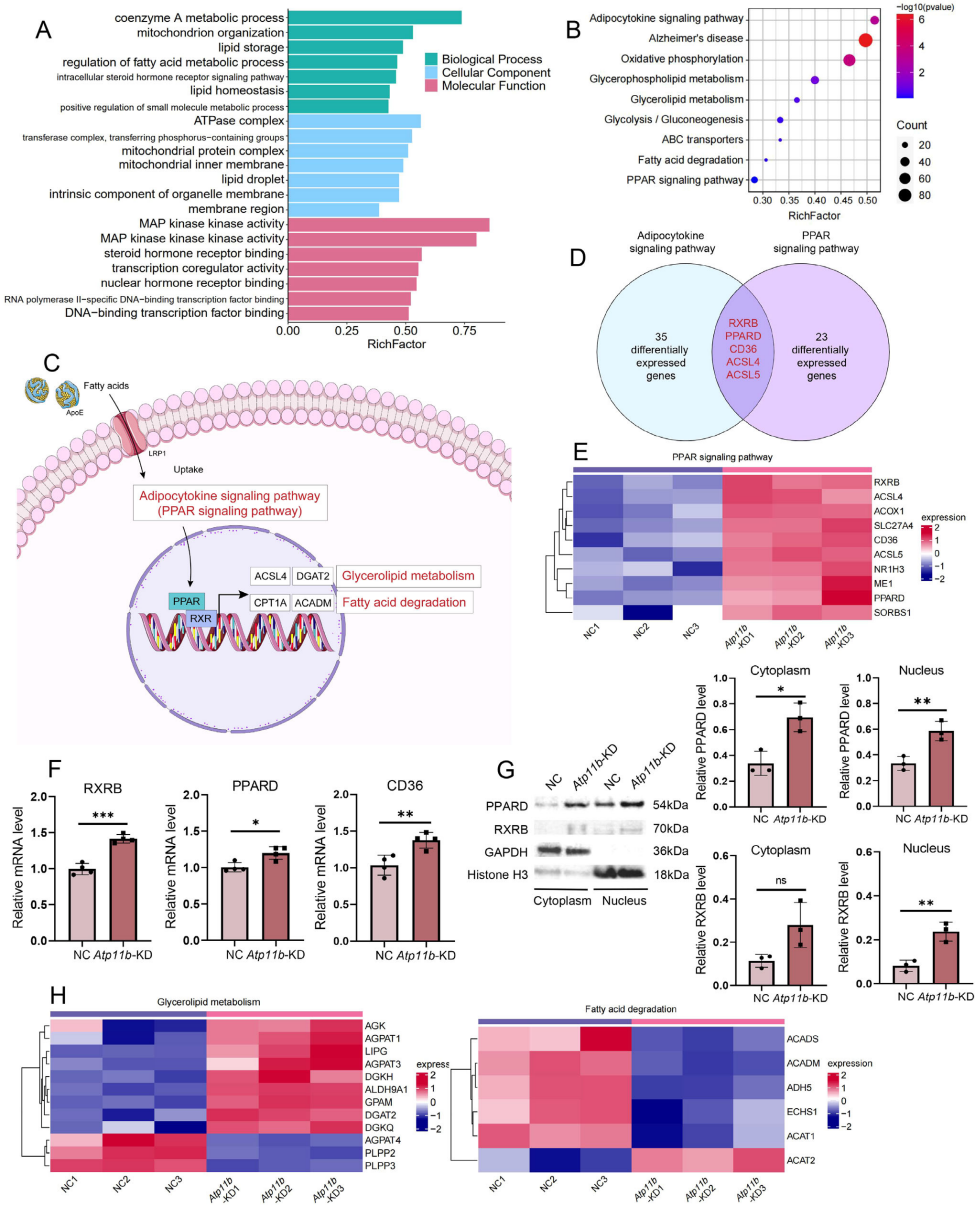
*Atp11b* silencing activates the PPAR signaling pathway in microglia. (A) GO analysis of NC and *Atp11b*‐KD BV2 cells. (B) KEGG enrichment analysis of NC and *Atp11b*‐KD BV2 cells. (C) Schematic representation of lipid metabolism‐related pathways in microglia. (D) DEGs identified in both adipocytokine signaling pathways and PPAR signaling pathways. (E) The expression of representative DEGs from PPAR signaling pathway in NC and *Atp11b*‐KD BV2 cells from the bulk RNA sequencing. (F) qPCR measurement of mRNA levels of RXRB, PPARD, and CD36 in NC and *Atp11b*‐KD BV2 cells. Two‐tailed Student's *t*‐test (*n* = 4 of two experiments). (G) Western blot images and quantification of PPARD and level in the nucleus and cytoplasm of NC and *Atp11b*‐KD BV2 cells. Two‐tailed Student's *t*‐test (*n* = 3 of two experiments). (H) The expression of representative DEGs from glycerolipid metabolism and fatty acid degradation in NC and *Atp11b*‐KD BV2 cells from the bulk RNA sequencing.

To investigate the impact of *Atp11b* deficiency on intracellular signaling pathways subsequent to lipid uptake, we conducted transcriptome sequencing on BV2 cells with *Atp11b*‐KD. Gene Ontology (GO) analysis revealed significant enrichment in pathways associated with lipid processes, encompassing coenzyme A metabolic process, lipid storage, and regulation of FA metabolic process (Figure 3A). Additionally, we investigated the impact of Atp11b deficiency on intracellular signaling pathways following lipid uptake by conducting transcriptome sequencing on BV2 cells with *Atp11b*‐KD. GO analysis revealed significant enrichment in pathways associated with lipid processes, including coenzyme A metabolic process, lipid storage, and regulation of FA metabolic processes (Figure 3A). Moreover, we observed transcriptional responses related to lipid signaling, such as the intracellular steroid hormone receptor signaling pathway, steroid hormone receptor binding, and transcription coregulator activity (Figure 3A). Kyoto Encyclopedia of Genes and Genomes (KEGG) analysis uncovered substantial regulatory effects of *Atp11b* on adipocytokine and peroxisome proliferator‐activated receptor (PPAR) signaling pathways (Figure 3B). These effects extended to downstream pathways associated with glycerolipid metabolism and FA degradation within the PPAR signaling cascade (Figure 3B).

Upon internalization of FAs, microglia act as ligands for PPARs, leading to their translocation into the cell nucleus. As a result, there is an increase in the transcription of proteins related to glycerolipid metabolism and FA degradation through the binding of PPAR to its nuclear receptor, retinoid X receptor (RXR) (Figure 3C) [40]. An intriguing partial overlap between the PPAR signaling pathway and the adipocytokine signaling pathway was discerned. Both pathways activated PPAR to orchestrate transcriptional regulation within the nucleus. Several key genes involved in both adipocytokine and PPAR signaling pathways were significantly upregulated in *Atp11b*‐KD BV2 cells, including RXRB, PPARD, CD36, ACSL4, and ACSL5 (Figure 3D,E). The upregulation of RXRB, PPARD, and CD36 in *Atp11b*‐KD BV2 cells was also confirmed by quantitative polymerase chain reaction (qPCR) (Figure 3F). Subcellular fractionation was performed to ascertain the nuclear translocation of PPARD and RXRB, which was confirmed by western blot (Figure 3G). RXRB in the nucleus of *Atp11b*‐KD BV2 cells was significantly increased and there was no change in the cytoplasm (Figure 3G). These results indicate that PPARD and RXRB enter the nucleus and play a role in regulating transcription. In summary, the effects of *Atp11b*‐KD on microglia extend to both glycerolipid metabolism and FA degradation pathways within the PPAR cascade.

Furthermore, critical genes associated with glycerolipid metabolism demonstrated increased expression, while those involved in FA degradation showed decreased expression (Figure 3H). This comprehensive regulatory network underscores the multifaceted impact of ATP11B on cellular lipid homeostasis. Further scrutiny is warranted to meticulously assess TG metabolism and FA degradation in *Atp11b*‐KD BV2 cells.

### 2.4 *Atp11b* Deficiency Increases Neutral Lipid Biogenesis and Decreases Lipolysis in Microglia

**FIGURE 4:**
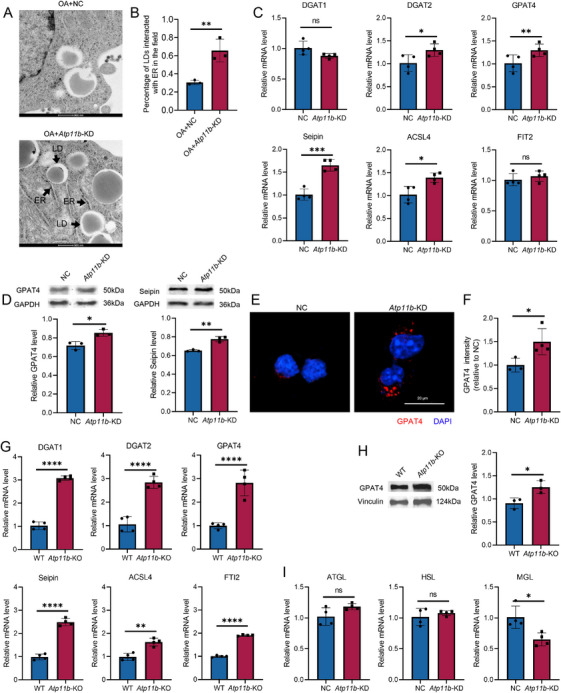
*Atp11b* deficiency increases neutral lipid biogenesis and decreases lipolysis in microglia. (A) Representative TEM images of LD interacted with ER in NC and *Atp11b*‐KD BV2 cells treated with OA (1 mM) for 12 h. (B) Quantification of the percentage of LDs interacted with ER in the field. Two‐tailed Student's *t*‐test (*n* = 3 of two experiments). (C) qPCR measurement of mRNA levels of proteins associated with TG synthesis (DGAT1, DGAT2, GPAT4, Seipin, ACSL4, and FIT2) in NC and *Atp11b*‐KD BV2 cells. Two‐tailed Student's *t*‐test (*n* = 4 of two experiments). (D) Western blot images and quantification of GPAT4 and Seipin level in NC and *Atp11b*‐KD BV2 cells. Two‐tailed Student's *t*‐test (*n* = 3 of two experiments). (E) Representative images of NC and *Atp11b*‐KD BV2 cells immunostained for GPAT4 and DAPI. (F) Quantification of GPAT4 intensity in BV2 cells. Two‐tailed Student's *t*‐test (*n* = 3 of two experiments). (G) qPCR measurement of mRNA levels of proteins associated with TG synthesis (DGAT1, DGAT2, GPAT4, Seipin, ACSL4, and FIT2) in microglia acute sorted from 6 M WT and *Atp11b*‐KO mice. Two‐tailed Student's *t*‐test (*n* = 4 of two experiments). (H) Western blot images and quantification of GPAT4 and Seipin level in microglia acute sorted from 6 M WT and *Atp11b*‐KO mice. Two‐tailed Student's *t*‐test (*n* = 3 of two experiments). (I) qPCR measurement of mRNA levels of enzymes associated with lipolysis (ATGL, HSL, and MGL) in NC and *Atp11b*‐KD BV2 cells. Two‐tailed Student's *t*‐test (*n* = 4 of two experiments).

The KEGG results revealed that *Atp11b* deficiency affected glycerolipid metabolism downstream of PPAR signaling pathway, so we further investigated whether *Atp11b* deficiency led to enhanced neutral lipid synthesis. We used transmission electron microscopy (TEM) to observe OA‐treated NC and *Atp11b*‐KD BV2 cells. The results showed that the interaction between LD and ER in *Atp11b*‐KD BV2 cells was obvious (Figure 4A,B). Since the ER is the site of neutral lipid synthesis, its contact with LDs indicates that excess neutral lipids are stored in the form of LDs.

We measured the mRNA levels of proteins associated with TG synthesis. qPCR assay showed that *Atp11b*‐KD induced the expression of diacylglycerol O‐acyltransferase 2 (DGAT2), glycerol‐3‐phosphate acyltransferase 4 (GPAT4), Berardinelli‐Seip congenital lipodystrophy 2 (Seipin), and acyl‐CoA synthetase long‐chain family member 4 (ACSL4) [41] (Figure 4C). However, the mRNA level of DGAT1 and fat storage‐inducing transmembrane protein 2 (FIT2) had no significant difference [41] (Figure 4C). Western blot assay also showed increased expression of GPAT4 and Seipin (Figure 4D). In addition, immunofluorescence images showed higher fluorescence intensity of GPAT4 in the cytoplasm of *Atp11b*‐KD BV2 cells (Figure 4E,F). Similarly, the expression levels of TG synthesis‐related proteins in microglia acutely isolated from 6 M WT and *Atp11b*‐KO mice were measured by qPCR. Results showed that the proteins detected in BV2 cells showed varying degrees of increase in *Atp11b*‐KO mouse microglia (Figure 4G). Moreover, the results of in vivo western blot assay for GPAT4 were the same as in vitro (Figure 4H). In conclusion, both in vivo and in vitro experiments confirmed that *Atp11b* deficiency led to increased neutral lipid synthesis in microglia.

In addition, the ability of BV2 cells to degrade ingested neutral lipids was examined. The lipolysis process involves three important lipases, namely adipose triglyceride lipase (ATGL), hormone‐sensitive lipase (HSL), and glycerol monoester lipase (MGL) [42, 43]. mRNA levels of ATGL and HSL did not change significantly after *Atp11b*‐KD but MGL decreased significantly (Figure 4I). In summary, *Atp11b* deficiency promoted neutral lipid accumulation by enhancing their biogenesis and inhibiting their degradation.

### 2.5 *Atp11b* Silencing Shifts Energy Metabolism From FAO to Glycolysis in Microglia

**FIGURE 5:**
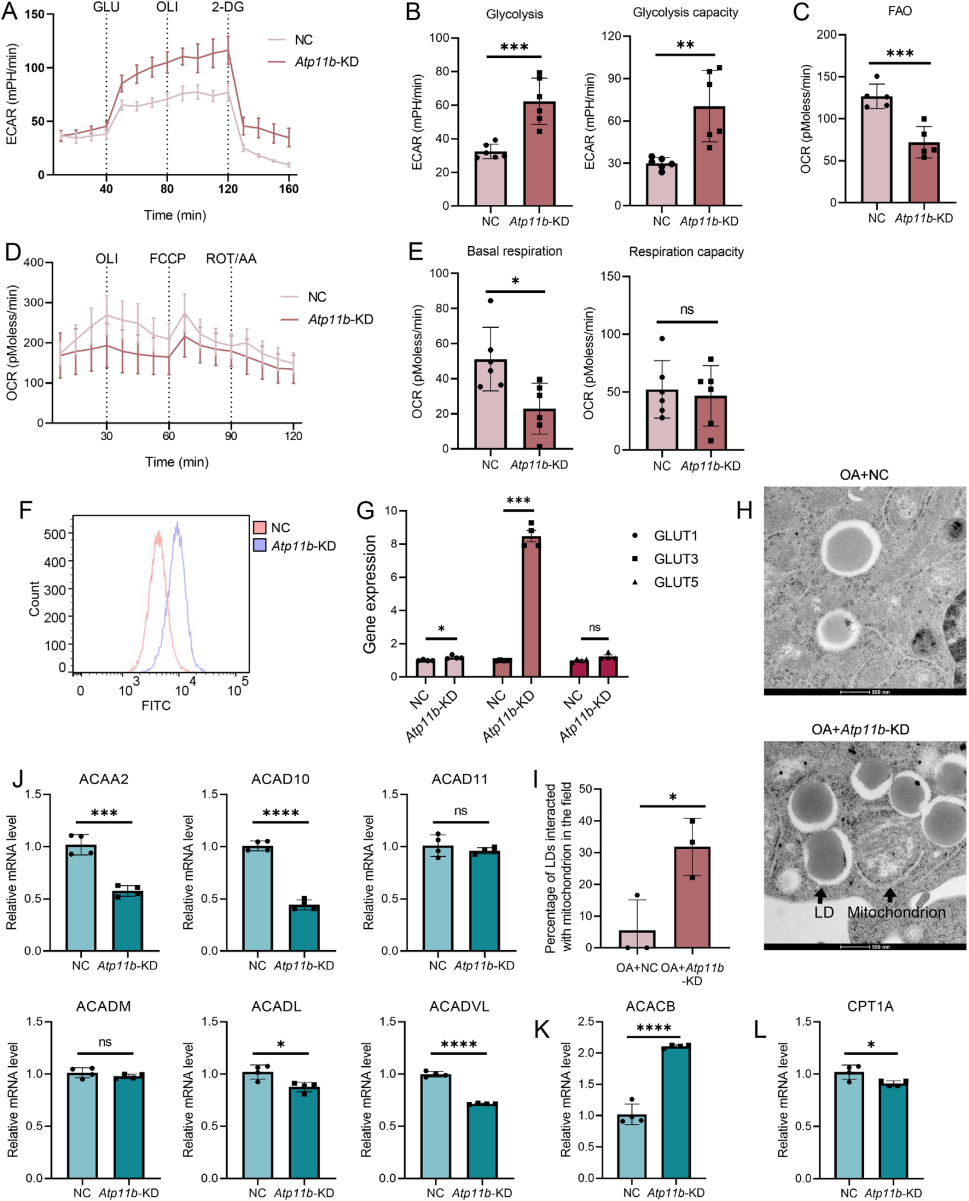
*Atp11b* silencing shifts energy metabolism from FAO to glycolysis in microglia. (A) Glycolysis level in NC and *Atp11b*‐KD BV2 cells reflected by ECAR. (B) Glycolysis and glycolysis capacity in BV2 cells. Two‐tailed Student's *t*‐test (*n* = 6 of two experiments). (C) FAO level in NC and *Atp11b*‐KD BV2 cells reflected by OCR. Two‐tailed Student's *t*‐test (*n* = 5 of two experiments). (D) Mitochondrial respiration in NC and *Atp11b*‐KD BV2 cells reflected by oxygen consumption rate (OCR). (E) Basal respiration and respiration capacity (the ratio of maximal to basal respiration) in BV2 cells. Two‐tailed Student's *t*‐test (*n* = 6 of two experiments). (F) Flow cytometry histogram of glucose uptake capacity in NC and *Atp11b*‐KD BV2 cells. Glucose uptake was measured by FITC fluorescence intensity. (G) qPCR measurement of mRNA levels of GLUT1, GLUT3, and GLUT5 in NC and *Atp11b*‐KD BV2 cells. Two‐tailed Student's *t*‐test (*n* = 4 of two experiments). (H) Representative TEM images of LD interacted with mitochondrion in NC and *Atp11b*‐KD BV2 cells treated with OA (1 mM) for 12 h. (I) Quantification of the percentage of LDs interacted with mitochondrion in the field. Two‐tailed Student's *t*‐test (*n* = 3 of two experiments). (J) qPCR measurement of mRNA levels of enzymes associated with FAO (ACAA2, ACAD10, ACAD11, ACADM, ACADL, and ACADVL) in NC and *Atp11b*‐KD BV2 cells. Two‐tailed Student's *t*‐test (*n* = 4 of two experiments). (K) qPCR measurement of mRNA levels of ACACB in NC and *Atp11b*‐KD BV2 cells. Two‐tailed Student's *t*‐test (*n* = 4 of two experiments). (L) qPCR measurement of mRNA levels of CPT1A in NC and *Atp11b*‐KD BV2 cells. Two‐tailed Student's *t*‐test (*n* = 4 of two experiments).

Since FA degradation and glycolysis were also enriched in KEGG analysis of *Atp11b*‐KD BV2 cells, we also explored the regulatory effect of ATP11B on energy metabolism in microglia. Extracellular acidification rate (ECAR) measurement showed that *Atp11b*‐KD enhanced the glycolysis and glycolysis capacity of BV2 cells (Figure 5A,B). However, *Atp11b*‐KD reduced FAO levels in BV2 cells (Figure 5C). Moreover, *Atp11b*‐KD BV2 cells exhibited lower basal respiration and respiratory capacity (the ratio of maximal to basal respiration) (Figure 5D,E). Since FAO produces more energy than glycolysis, the transformation of energy metabolism induced by *Atp11b*‐KD is one reason for the decreased respiratory ability of BV2 cells [44]. Besides, after adding oligomycin (OLI) to inhibit oxidative phosphorylation in BV2 cells, *Atp11b*‐KD led to a compensatory increase in glycolysis rate in BV2 cells (Figure 5A), which also confirmed that *Atp11b*‐KD led to a decrease in the aerobic metabolism mode of FAO in BV2 cells, thereby increasing compensatory glycolysis. Flow cytometry confirmed that *Atp11b*‐KD enhanced the glucose uptake capacity in BV2 cells (Figure 5F). In addition, *Atp11b*‐KD BV2 cells showed elevated expressions of glucose transporter (GLUT) 1 and GLUT3 (Figure 5G). These results support the conclusion that *Atp11b*‐KD enhances glucose utilization by BV2 cells and modulates the energy metabolism pathway toward glycolysis.

In addition, we observed increased contact between mitochondria and LDs under OA treatment using TEM in *Atp11b*‐KD BV2 cells (Figure 5H,I), possibly indicating mitochondria supporting LD expansion [45, 46]. The mRNA levels of FAO‐related enzymes were detected by qPCR. The expressions of acetyl‐CoA acyltransferase 2 (ACAA2), acyl‐CoA dehydrogenase family member 10 (ACAD10), acyl‐CoA dehydrogenase long‐chain (ACADL), and acyl‐CoA dehydrogenase very long chain (ACADVL) in *Atp11b*‐KD BV2 cells decreased in different degrees (Figure 5J). There was no change in the expression of ACAD11 and acyl‐CoA dehydrogenase medium chain (ACADM) (Figure 5J). The expression of acetyl‐CoA carboxylase β (ACACB) that negatively regulated FAO was increased (Figure 5K). In addition, *Atp11b*‐KD BV2 cells also decreased the expression of carnitine palmitoyl transferase 1a (CPT1A), which transfers fatty acyl‐Coenzyme A into mitochondria (Figure 5L). In conclusion, *Atp11b*‐KD decreased the FAO levels of BV2 cells suggesting reduced lipid utilization.

### 2.6 *Atp11b* Deficiency Leads to Microglial LD Accumulation That Can be Alleviated by *Atp11b* Overexpression

**FIGURE 6:**
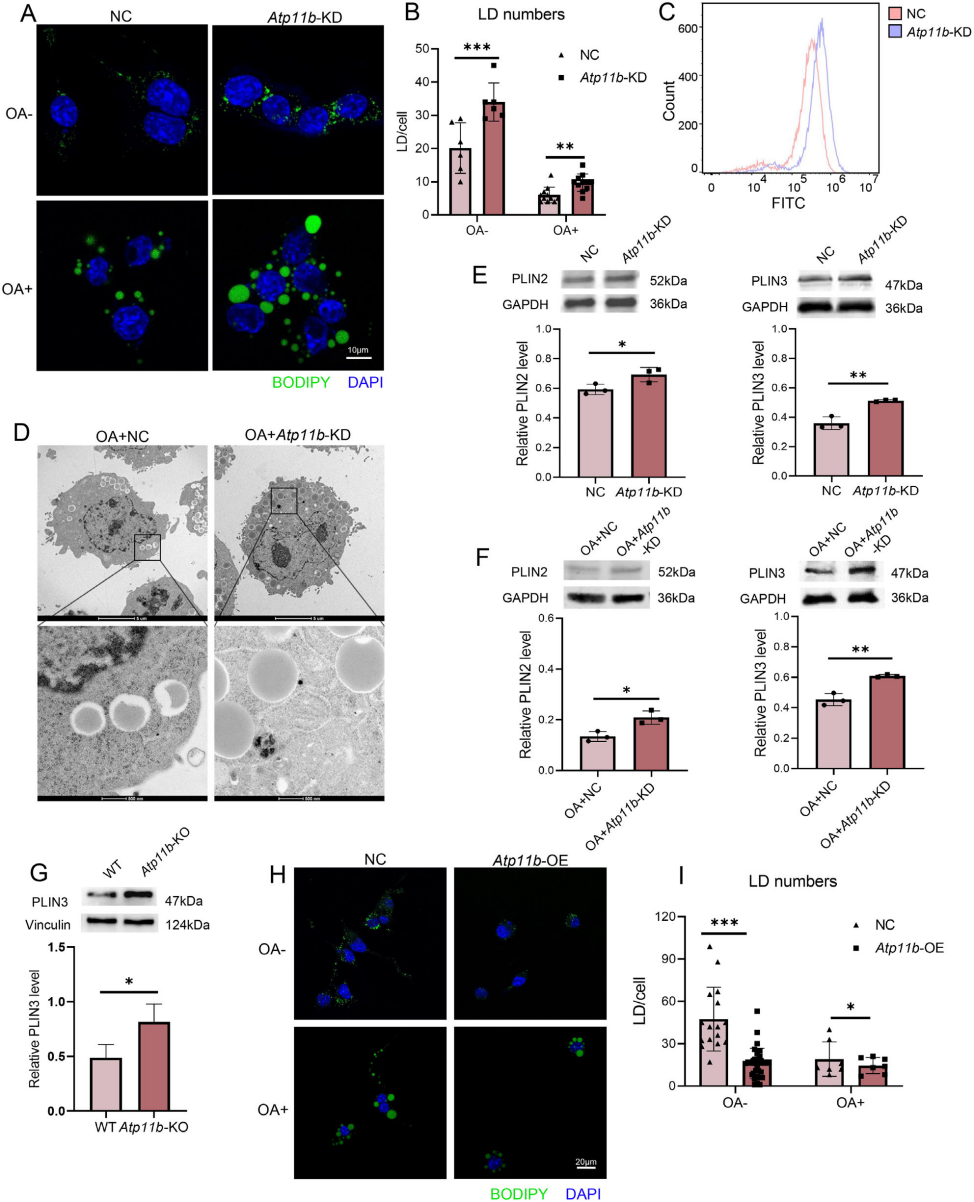
*Atp11b* deficiency leads to microglial LD accumulation that can be alleviated by *Atp11b* overexpression. (A) Representative images of NC and *Atp11b*‐KD BV2 cells treated with OA (1 mM) for 12 h immunostained for BODIPY (LDs) and DAPI. (B) Quantification of BODIPY+ LD numbers of A. One‐way ANOVA (*n* = 3). (C) Flow cytometry histogram of BODIPY mean fluorescence intensity in NC and *Atp11b*‐KD BV2 cells. (D) Representative TEM images of LD structures in NC and *Atp11b*‐KD BV2 cells treated with OA (1 mM) for 12 h. (E and F) Western blot images and quantification of PLIN2 and PLIN3 levels in NC and *Atp11b*‐KD BV2 cells (E) and treated with OA (1 mM) for 12 h (F). Two‐tailed Student's *t*‐test (*n* = 3 of two experiments). (G) Western blot images and quantification of PLIN3 level in microglia acute sorted from 6 M WT and *Atp11b*‐KO mice. Two‐tailed Student's *t*‐test (*n* = 3 of two experiments). (H) Representative images of NC and *Atp11b*‐OE BV2 cells treated with PBS or OA (1 mM) for 12 h immunostained for BODIPY (LDs) and DAPI. (I) Quantification of BODIPY+ LD numbers in H. Two‐tailed Student's *t*‐test (*n* = 3 of two experiments).

The increase of neutral lipids and the decrease of FAO levels in microglia caused by *Atp11b* deficiency both suggested microglia decrease the utilization of FAs and choose to enhance lipid storage. Therefore, we investigated whether *Atp11b* deficiency microglia had more LDs. Immunofluorescence results showed that the number of LDs in *Atp11b*‐KD BV2 cells was more than in NC BV2 cells without or with OA treatment (Figure 6A,B). Flow cytometry measurements of BODIPY fluorescence intensity supported this observation (Figure 6C). Furthermore, TEM analysis demonstrated that *Atp11b*‐KD BV2 cells possessed larger LDs containing more neutral lipids (Figure 6D). In addition, we quantified the level of perilipin (PLIN) by western blotting. The results of in vitro experiments showed that the levels of PLIN2 and PLIN3 in *Atp11b*‐KD BV2 cells were increased without or with OA treatment (Figure 6E,F). In vivo, results also showed elevated PLIN3 levels in the microglia of *Atp11b*‐KO mice (Figure 6G) compared with WT mice. Having confirmed that *Atp11b* deficiency leads to LD accumulation in microglia, we proceeded to investigate the potential therapeutic role of ATP11B in this pathological phenomenon. We transfected BV2 cells with lentivirus containing human *Atp11b* sequence (hereinafter referred to as *Atp11b*‐overexpression (*Atp11b*‐OE)). The fluorescence intensity of the red fluorescent protein (RFP) carried by lentivirus reflected ATP11B in BV2 cells (Figure S3B). qPCR results also showed that the mRNA of *Atp11b* in BV2 cells transfected with lentivirus increased significantly (Figure S3C). Immunofluorescence results showed that *Atp11b*‐OE significantly reduced the number of LDs in BV2 cells in both untreated and OA‐treated conditions (Figure 6H,I). Collectively, these results suggest that ATP11B may have a therapeutic effect on pathological LD accumulation.

### 2.7 Overexpression of *Atp11b* Suppresses AD Microglial LD Accumulation both in Vivo and in Vitro

**FIGURE 7:**
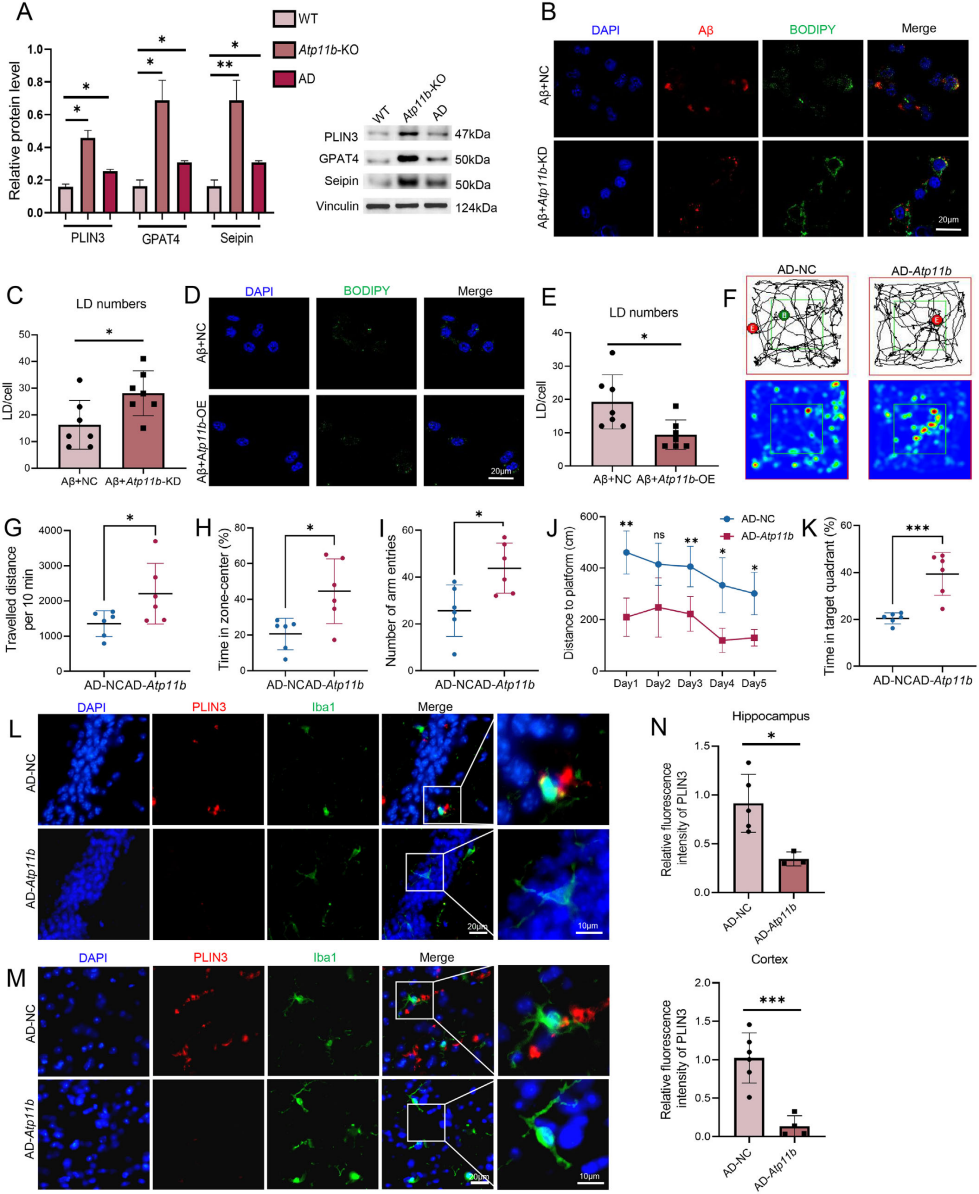
Overexpression of *Atp11b* suppresses AD microglial LD accumulation both in vivo and in vitro. (A) Western blot images and quantification of PLIN3, GPAT4, and Seipin level in microglia acute sorted from 9 M WT, *Atp11b*‐KO, and AD mice. One‐way ANOVA. (B) Representative images of NC and *Atp11b*‐KD BV2 cells both treated with Aβ (TAMRA) for 24 h immunostained for BODIPY (LDs) and DAPI. (C) Quantification of BODIPY+ LD numbers of NC and *Atp11b*‐KD BV2 cells both treated with Aβ (TAMRA) for 24 h. Two‐tailed Student's *t*‐test (*n* = 3 of two experiments). (D) Representative images of NC and *Atp11b*‐OE BV2 cells both treated with Aβ for 24 h immunostained for BODIPY (LDs) and DAPI. (E) Quantification of BODIPY+ LD numbers of NC and *Atp11b*‐OE BV2 cells both treated with Aβ for 24 h. Two‐tailed Student's *t*‐test (*n* = 3 of two experiments). (F) Trajectory diagram of the route of AD‐NC and AD‐*Atp11b* mice in the open field test. (G) Traveled distance per 10 min of AD‐NC and AD‐*Atp11b* mice in the open field test. Two‐tailed Student's *t*‐test (*n* = 6 of two experiments). (H) The percentage of the time in the zone‐center of AD‐NC and AD‐*Atp11b* mice in the open field test. Two‐tailed Student's *t*‐test (*n* = 6 of two experiments). (I) The number of arm entries of AD‐NC and AD‐*Atp11b* mice in Y maze test. (J) Distance to the platform of AD‐NC and AD‐*Atp11b* mice during train days in Morris water maze experiment. (K) Time in target quadrant of AD‐NC and AD‐*Atp11b* mice during probe trial in Morris water maze experiment. (L) Representative images of the hippocampus of AD‐NC and AD‐*Atp11b* mice immunostained for PLIN3 (LDs), Iba1 (microglia), and DAPI. (M) Representative images of the cortex of AD‐NC and AD‐*Atp11b* mice immunostained for PLIN3 (LDs), Iba1 (microglia), and DAPI. (N) Quantification of L and M. Two‐tailed Student's *t*‐test (*n* = 3 of two experiments).

Since ATP11B has been shown to alleviate the accumulation of LDs in microglia in a nonpathological state, we further investigated whether ATP11B can suppress the accumulation of LDs in microglia in AD state. Western blot analysis of microglia isolated from AD mice showed increased protein levels associated with LD accumulation, thus confirming the phenomenon of LD accumulation in AD microglia (Figure 7A). The in vitro model of AD was constructed by treating BV2 cells with Aβ1‐42 (bestbiochem, 5‐TAMRA labeled, human) for 24h. *Atp11b*‐KD increased the accumulation of LDs in BV2 cells treated with Aβ (Figure 7B,C). While *Atp11b*‐OE decreased the accumulation of LDs in BV2 cells treated with Aβ (Figure 7D,E). Western blot results confirmed that ATP11B increased significantly in *Atp11b*‐OE BV2 cells treated with Aβ (Figure S3D). Then, we investigated the alleviation of *Atp11b* overexpression on LD accumulation in AD mice. We injected lentivirus containing Cx3cr1 and human *Atp11b* sequence using brain stereotaxic injection into the hippocampus of six AD mice at the age of 6 months and evaluated the exploratory behavior 1 month later. qPCR results showed that the mRNA of *Atp11b* in acutely isolated microglia increased significantly in AD mice injected with *Atp11b* lentivirus (Figure S3E). Open field experiment indicated AD mice injected with *Atp11b* had more active exploratory behavior reflected by more time and distance in the zone‐center (Figure 7F–H). The Y Maze experiment showed that AD mice injected with *Atp11b* had an increased number of total arm entries, indicating enhanced exploratory behavior and memory ability (Figure 7I). Moreover, AD mice injected with *Atp11b* displayed enhanced learning and memory abilities, as evidenced by covering a shorter distance when finding the platform during training days and increased time spent in the target quadrant during probe trials in the Morris water maze experiment (Figure 7J,K). Immunofluorescence results of brain sections showed that ATP11B alleviated the accumulation of LDs in the hippocampus and cortex of AD mice (Figure 7L–N). In conclusion, both in vitro and in vivo results showed that ATP11B suppressed the accumulation of LDs in AD.

### 2.8 Overexpression of *Atp11b* Alleviates Aβ Accumulation and Reduces Inflammatory Factors in AD Mice

**FIGURE 8:**
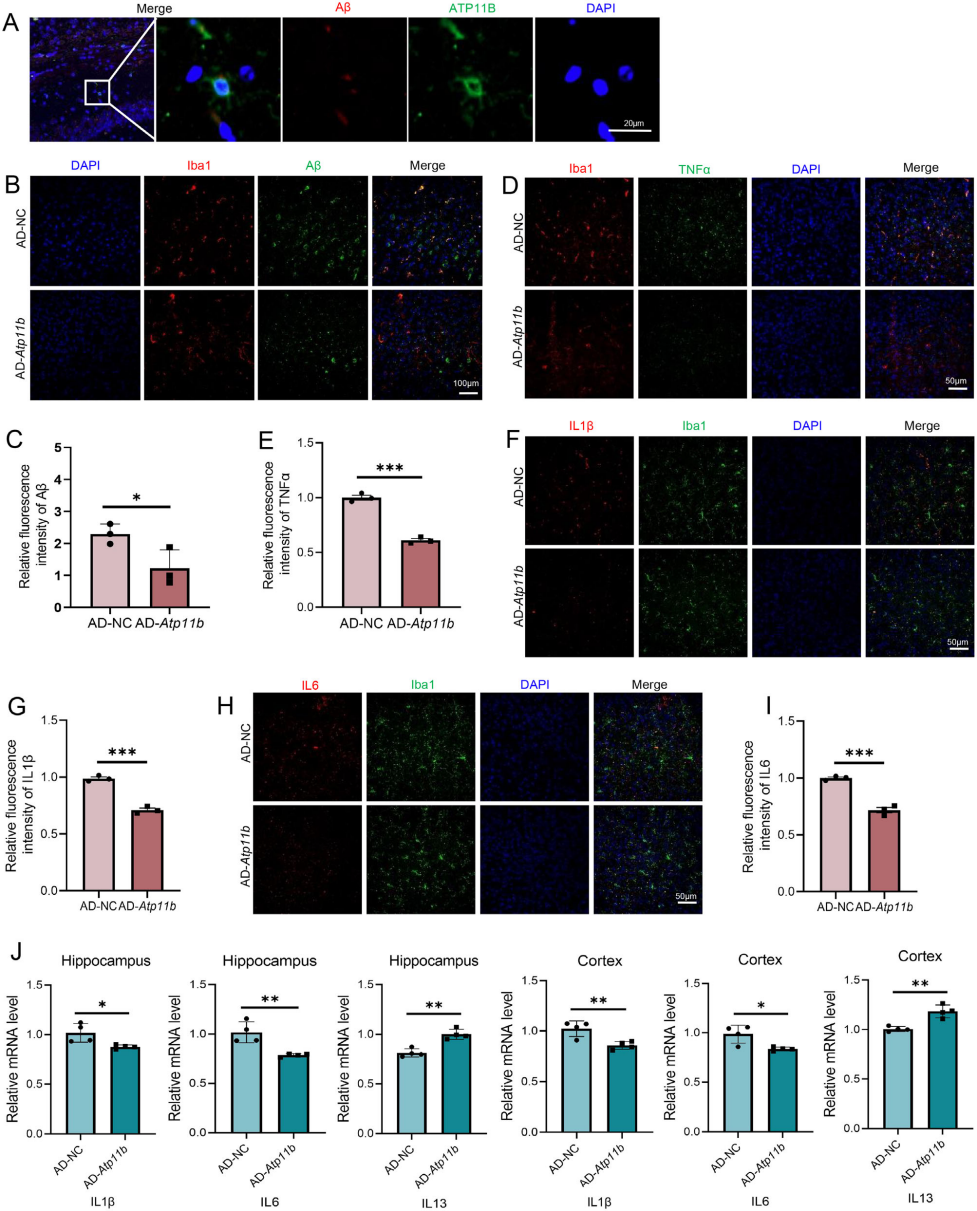
Overexpression of *Atp11b* alleviates Aβ accumulation and reduces inflammatory factors in AD mice. (A) Representative images of the immunofluorescence colocalization of Aβ with ATP11B in the cortex of AD mouse. (B) Representative images of the cortex of AD‐NC and AD‐*Atp11b* mice immunostained for Iba1 (microglia), Aβ, and DAPI. (C) Quantification of B. (D) Representative images of the cortex of AD‐NC and AD‐*Atp11b* mice immunostained for Iba1 (microglia), TNFα, and DAPI. (E) Quantification of D. Two‐tailed Student's *t*‐test (*n* = 3 of two experiments). (F) Representative images of the cortex of AD‐NC and AD‐*Atp11b* mice immunostained for Iba1 (microglia), IL1β, and DAPI. (G) Quantification of F. Two‐tailed Student's *t*‐test (*n* = 3 of two experiments). (H) Representative images of the cortex of AD‐NC and AD‐*Atp11b* mice immunostained for Iba1 (microglia), IL6, and DAPI. (I) Quantification of H. Two‐tailed Student's *t*‐test (*n* = 3 of two experiments). (J) qPCR measurement of mRNA levels of IL1β, IL6, and IL13 in the hippocampus and cortex of AD‐NC and AD‐*Atp11b* mice. Two‐tailed Student's *t*‐test (*n* = 4 of two experiments).

We further investigated the ameliorative effect of ATP11B on other pathological AD phenomena. We injected *Atp11b* lentivirus using brain stereotaxic injection into the hippocampus of six AD mice at the age of 6 months and evaluated the AD pathology 1 month later. We observed the colocalization of ATP11B and Aβ (Figure 8A). Immunofluorescence results showed that ATP11B treatment reduced Aβ plaques in the cortex of AD mice (Figure 8B,C). These results suggested that ATP11B restored the activity of microglia in AD mice and enhanced phagocytosis and clearance. Tumor necrosis factor (TNFα), interleukin 1β (IL1β), and IL6 were highly expressed in the cortex of AD mice, while the expression of these inflammatory cytokines was significantly reduced after ATP11B treatment (Figure 8D–I). qPCR results also showed that ATP11B decreased IL1β and IL6 and increased IL13 in the hippocampus and cortex of AD mice (Figure 8J). This suggested that the chronic activation of microglia was alleviated after overexpression of *Atp11b* and the phenomenon of abnormal secretion of inflammatory factors caused by overactivation was also improved. Overall, treatment of ATP11B significantly improved pathological conditions in AD mice, including alleviating Aβ plaque deposition and alleviating inflammatory responses in the brain microenvironment.

## 3 Discussion

Here, we discovered that the absence of *Atp11b* resulted in a disruption of lipid metabolism in microglia. This deficiency led to an excessive uptake of FAs, consequently activating the PPAR signaling pathway, which in turn led to abnormal synthesis of neutral lipids and mitochondrial energy metabolism in microglia. Subsequent findings revealed the accumulation of pathological LDs in both microglia and mice with AD as a result of *Atp11b* deficiency. Additionally, upregulation of *Atp11b* mitigated LD accumulation, Aβ deposition, inflammatory responses, exploratory behavior impairment, and learning and memory impairment in AD mice. Our hypothesis suggests that ATP11B plays a crucial role in maintaining the balance of intracellular lipid composition through the regulation of intracellular lipid transport in the CNS. Consequently, ATP11B presents a promising avenue for the development of treatments for neurological diseases, such as AD.

The changes in lipid composition and metabolism in microglia may be due to the response to the external environment. *Atp11b* deficiency may lead microglia to excessive uptake of lipids excreted by neurons and other glial cells via the lipid uptake pathway associated with ApoE and LRP1 in the CNS [37]. As a result, the lipid load of microglia increases even beyond their capacity. Also, the lipid uptake pathway associated with ApoE and LRP1 is a pathway for microglia to phagocytize Aβ [47]. Identifying the primary cause of the increase in ApoE and LRP1 caused by *Atp11b* deficiency may help to address the fundamental issue of LD accumulation and the development and progression of AD.

*Atp11b* deficiency may potentially disrupt lipid metabolic pathways by impacting the membrane proteins of organelles such as the ER. The final step of TG synthesis in the ER is catalyzed by DGAT and DGAT1 is mainly responsible for the formation of TGs from the extra lipids taken up by microglia to avoid the ER stress caused by toxic lipids [1, 41]. In addition, Seipin plays a decisive role in the localization of neutral lipids in the ER and the process of LDs separating from the ER to form independent organelles [48]. *Atp11b* deficiency significantly increased the number of these two membrane proteins, suggesting a potential interaction with ATP11B on the ER cell membrane to mediate the synthesis of neutral lipids and the formation of LDs.

The alteration in energy metabolism may serve as a mechanism for self‐regulation and protection in microglia. *Atp11b* deficiency resulted in a shift in the primary energy source of microglia from FAs to glucose, and a switch from FAO to glycolysis as the primary mode of energy metabolism. Microglial energy metabolism demonstrates great flexibility, adapting to mitochondrial dysfunction and increasing intracellular ROS by forming LDs to prevent ROS‐induced lipid peroxidation [7, 49]. This shift is crucial, as unsaturated FAs contained within LDs are more resistant to oxidation than those found in the membrane [50]. Downregulation of FAO may occur in response to *Atp11b* deficiency to safeguard cells from ROS accumulation and potential impairment of microglial energy metabolism.

Furthermore, deficiency in *Atp11b* has been found to cause changes in mitochondrial function, leading to a reduction in adenosine triphosphate (ATP) levels. This, in turn, impacts the energy supply within microglia. Such disruption in energy metabolism may exacerbate the accumulation of pathological LD in microglia, thus contributing to the development of AD. Interestingly, both aging microglia and disease‐associated microglia (DAM) exhibit LD accumulation and enhanced glucose metabolism [18, 51]. The metabolic profile of *Atp11b*‐deficient microglia resembles that of DAM, possibly due to the enhancement of inflammatory responses and subsequent microglial activation caused by *Atp11b* deficiency. The relief of brain inflammation in AD mice by ATP11B further supports this hypothesis. While ATP11B is known to regulate lipid metabolism and the expression of inflammatory factors in microglia, it remains uncertain whether the overexpression of *Atp11b* can inhibit microglial activation by improving lipid metabolism. This perspective offers a means to suppress the inflammatory response of DAM by addressing lipid metabolism from its core.

Targeting the LD accumulation of microglia may offer a new approach to treating neurodegenerative diseases. Overexpression of *Atp11b* reduced abnormal accumulation of pathological LDs and alleviated the development of AD. The regulatory targets of ATP11B may be involved in lipid metabolism pathways both related to LD formation and AD. Such as ApoE4 and TREM2, two AD risk genes, are involved in the transport and uptake of extracellular lipids and can exacerbate the pathological phenomenon of LD accumulation [52, 53]. Besides, ApoE4 and TREM2 also can directly bind Aβ to help microglia phagocytosis Aβ and alter the activation of microglia [37, 54]. Understanding how AD risk genes regulate lipid uptake and LD accumulation is crucial for developing treatment strategies to address pathological LD accumulation in AD. It remains to be investigated whether ATP11B exerts its therapeutic effects on pathological LDs primarily through signaling pathways associated with ApoE4 and TREM2, ultimately improving cognitive impairment and AD pathology in AD mice. Further research in this area may provide valuable insights into the role of microglial lipid metabolism in AD pathogenesis and lead to the development of more effective treatment approaches, offering hope for AD patients.

## 4 Conclusions

Our study revealed that *Atp11b* deficiency led to the accumulation of pathological LDs in microglia and exacerbated the activation of Aβ aggregation and inflammatory responses by altering lipid metabolism in microglia. This disruption included changes in lipid composition, synthesis, degradation, uptake, and mitochondrial energy metabolism. Overexpression of *Atp11b* reversed these adverse effects, offering a new approach to addressing AD. Currently, the specific research on ATP11B related to treating abnormal lipid metabolism is still in its early stages, and multiple factors and molecular mechanisms may remain to be investigated.

## 5 Materials and Methods

### 5.1 Cell Culture

Murine microglial cell line BV2 cells were purchased from Hunan Fenghui Biotechnology Co., Ltd. (Hunan, China). BV2 cells were kept in liquid nitrogen in a rapid cell freezing medium (Life‐iLab, Shanghai, China; AC05L033). The cells were resuscitated and passaged two or three times before use. BV2 cells were cultured in DMEM (Biological Industries; 06‐1055‐57‐1A) supplemented with 10% fetal bovine serum (Gibco; 10099141) in a 5% CO_2_ incubator at 37 °C.

### 5.2 Transfection

Lipofectamine™ 2000 (Invitrogen; 11668019) reagent and siRNA were diluted respectively using Opti‐MEM™ medium (Gibco; 31985070) before siRNA transfection and the two diluents were mixed and added into the culture medium of BV2 cells. The cells were incubated at 37°C for 1–2 days before follow‐up cell experiments. The lentivirus solution was diluted in the culture medium according to the MOI (100TU) of BV2 cells. After 48 h, add puromycin (Yeasen Biotechnology Co., Ltd., Shanghai, China) to sort for stable expression of *Atp11b* cell lines. siRNA and lentivirus were purchased from Beijing Tsingke Biotech Co., Ltd. (Beijing, China). The siRNA sequence targeting *Atp11b* was 5′‐GGACACUGUGUAUAGCUUA(dT)(dT)‐3′ against 5′‐UAAGCUAUACACAGUGUCC (dT)(dT)‐3′.

### 5.3 Microglia Acutely Isolation

Hippocampal and cortical tissues were dissected from the brains of mice. The tissues were incubated with Hanks’ balanced salts solution containing 0.025% trypsin (Gibco; 25200072) at 37°C for 20 min. Single‐cell suspension was obtained using a 40 µm cell filter. CD11b Microbeads (Miltenyi Biotech; 130‐093‐634) were added to label microglia. The microglia were isolated and collected using the MACS separator (Miltenyi Biotech; 130‐042‐102). The collected microglia were used for subsequent experimental analysis.

### 5.4 Animals

All mice were maintained in SPF animal laboratories at Shanghai University. Mice were reared at a constant temperature (22 ± 1°C) and a 12 h/12 h light/dark cycle provided enough food and water. C57BL/6 mice were purchased from Shanghai Model Organisms Center, Inc. (Shanghai, China). *Atp11b* ^−/−^ mice were constructed by Beijing Viewsolid Biotechnology Co. Ltd. (Beijing, China) using CRISPR technology. 3×Tg‐AD mice were purchased from The Jackson Laboratory. The 3×Tg‐AD mice treated with control lentivirus and treated with *Atp11b* lentivirus groups each consisted of 10 mice, five males and five females aged 6 months. However, due to mortality occurring during the stereotaxic injection procedure, only six mice per group were ultimately used for the behavioral testing. The mice were treated by the National Research Council's Guide for the Care and Use of Laboratory Animals and the study design was approved by the Animal Ethics Committee of Shanghai University (Approval No. ECSHU 2023–028, Date: June 13, 2023).

### 5.5 Immunofluorescence and Immunohistochemistry

BV2 cells were fixed with a PBS solution containing 5% paraformaldehyde. BV2 cells and brain tissue sections were treated with PBS containing 10% goat serum for 30 min. Then, primary antibodies were added and maintained at 4°C for 24 h. Primary antibodies: anti‐ATP11B (Invitrogen; PA5‐20996), anti‐IBA1 (Abcam; ab178846), anti‐TOM20 (ABclonal; A19403), anti‐COX4 (ABclonal; A11631), anti‐LAMP2 (ABclonal; A0593), anti‐GPAT4 (ABclonal; A20804), anti‐TIP47 (Santa Cruz Biotechnology; sc‐390968), anti‐Aβ (Cell signaling Technology; #8243), anti‐TNFα (ABclonal; A11534), anti‐IL1β (proteintech; 16806‐1‐AP), and anti‐IL6 (ABclonal; A0286). Then, the cells were treated with secondary antibodies at room temperature for 2 h. Secondary antibodies: goat anti‐rabbit (Alexa Fluor 488) (Abcam; ab150077), goat anti‐rabbit (Alexa Fluor 594) (Abcam; ab150080), goat anti‐mouse (Alexa Fluor 488) (ab150113), and goat anti‐mouse (Alexa Fluor 594) (ab150116). After washing with PBS, incubated with DAPI staining solution (Beyotime Biotechnology, Shanghai, China; C1005) for 15 min. Imaging was captured using a Zeiss LSM710 microscope.

### 5.6 BODIPY Staining

BODIPY 493/503 (Invitrogen; D3922) was configured as 1 mg mL^−1^ storage solution using dimethyl sulfoxide. BV2 cells were fixed with a PBS solution containing 5% paraformaldehyde. BODIPY storage solution was diluted to 1 µg mL^−1^ by PBS and added to BV2 cells for 5 min. BV2 cells were washed with PBS and incubated with a DAPI staining solution for 15 min. Images of BODIPY+ BV2 cells were captured using a Zeiss LSM710 microscope.

### 5.7 Lipid UPLC‐QE MS Analysis

BV2 cells were treated with DMEM containing 1 mM OA for 12 h. BV2 cells were extracted in methyl tert‐butyl ether (MTBE)/methanol/water (v/v/v, 10:2:5) contained with 2 µg mL^−1^ IS (LPC 17:0) to obtain the lipids. After vortex‐mixed for 60 s, the mixed samples were sonicated for 15 min and then placed at room temperature for 5 min. After centrifuging at 13,000×*g* for 15 min at 4°C, 400 µL of MTBE was transferred to an Eppendorf tube and evaporated under nitrogen. The dried extracts were reconstituted with 100 µL of dichloromethane/methanol (v/v, 1:1) solution and transferred to auto‐sampler vials. UPLC‐QE MS analysis was performed on a Vanquish HPLC system (Thermo Scientific, Germany) equipped with a Q‐Exactive Plus Orbitrap mass spectrometer (Thermo Scientific). Chromatographic separations were performed on a Waters XBridge^TM^ BEH C18 column (2.1 mm × 100 mm, 2.5 µm; Waters, Milford, MA).

### 5.8 TG, FC, and FFA Content Assay

TG content assay kit (AKFA003 M), FC content assay kit (AKFA001 M), and FFA content assay kit (AKFA008 M) were purchased from Beijing Boxbio Science and Technology Co., Ltd. (Beijing, China). BV2 cells were broken by ultrasound and the supernatant was obtained by centrifugation. Then, the instructions of different kits were followed to add the corresponding reagents. The absorbance was measured at OD 420 nm, OD 500 nm, and OD 550 nm to calculate the content of TG, FC, and FFA, respectively.

### 5.9 BODIPY 558/568 C12 Uptake Assay

BV2 cells were incubated with 1 mM BODIPY 558/568 C12 in DMEM for 18 h and fixed with a solution containing 5% paraformaldehyde. BV2 cells were incubated with the DAPI staining solution for 15 min. Images of BODIPY+ BV2 cells were captured using a Zeiss LSM710 microscope.

### 5.10 RNA Extraction and Sequencing

BV2 cells were incubated with TRIzol at a ratio of 2 × 10^6^ cells plus 1 mL TRIzol. Chloroform was added and maintained at room temperature for 10 min, after which it was centrifuged at 15,000×*g*  for 15 min at 4°C. The upper phase was transferred to a new tube and added 600 µL of isopropanol and recovered by centrifugation at 15,000×*g* for 30 min at 4°C. The RNA pellet was washed three times with 600 µL of 75% ethanol and treated with DNAse I. Then, the RNA was repurified with RNeasy Protect Mini Kit (Qiagen) according to the manufacturer's instructions and eluted in 50 µL of RNase‐free water. Illumina HiSeq‐compatible library was constructed with ScriptSeq Complete (Human/Mouse/Rat) library preparation kit (Epicentre). The quality and size distribution of sequencing libraries was analyzed with Agilent BioAnalyzer 2100.

### 5.11 Hippocampal Stereotaxic Injection

3×Tg mice at 6 months of age were anesthetized with isoflurane and mounted on a stereotaxic frame. The site of hippocampal injection was determined according to Franklin and Paxinos mouse brain atlas (anteroposterior 1.95 mm, mediolateral ±1.5 mm, dorsoventral 1.85 mm). Lentivirus containing human *Atp11b* sequence (3 µL, 4.0 × 10^8^ TU mL^−1^) was injected into the hippocampus of mice using a Hamilton needle.

### 5.12 Western Blot

BV2 cells or acutely sorted microglia were homogenized in precooled RIPA Lysis Buffer (Yeasen Biotechnology Co., Ltd.) and lysed for 30 min in an ice bath. The samples were centrifuged at 15,000×*g* for 10 min. The supernatant was collected and the protein concentration was determined. The protein was mixed with loading buffer and separated by sodium dodecyl sulfate‐polyacrylamide gel electrophoresis. This was subsequently transferred to a polyvinylidene difluoride membrane. After incubation with primary antibodies overnight and secondary antibodies for 2 h, the membranes were developed by the ChemDoc MP Imaging System (BIO‐RAD). Primary antibodies: anti‐vinculin (Abways; CY5164), anti‐LRP1 (ABclonal; A0633), anti‐APOE (proteintech; 18254‐1‐AP), anti‐GAPDH (ABclonal; AC002), anti‐LPL (ABclonal; A16252), anti‐GPAT4 (ABclonal; A20804), anti‐BSCL2 (ABclonal; A14583), anti‐PLIN‐2 (ABclonal; A20843), and anti‐PLIN3 (ABclonal; A15776). Second antibodies: HRP‐conjugated goat anti‐mouse (proteintech; SA00001‐1), HRP‐conjugated goat anti‐rabbit (proteintech; SA00001‐2), anti‐mouse IgG DyLight 680‐Labeled (seracare; 5230‐0406), and anti‐rabbit IgG DyLight 800‐Labeled (seracare; 5230‐0412).

### 5.13 Reverse Transcription Quantitative Polymerase Chain Reaction

Total RNA was isolated from BV2 cells or acutely sorted microglia using RNA extraction solutions (Servicebio; G3013, Wuhan, China). RNA quality was evaluated by NanoDrop One Microvolume UV‐Vis Spectrophotometer (Thermo Fisher). The extracted RNA was reverse transcribed into cDNA using ABScript III RT Master Mix for qPCR with gDNA Remover (ABclonal; RK20429, Wuhan, China). qPCR was performed by mixing the cDNA template, primers, and 2× Universal SYBR Green Fast qPCR Mix (ABclonal; RK21203) according to the procedure. Comparative Ct (ΔΔCt) method was used to calculate the relative target gene expression level compared with the reference group.

### 5.14 LPL Activity Assay

LPL activity assay kit (AKFA012 M) was purchased from Beijing Boxbio Science and Technology Co., Ltd. Then, the corresponding reagent was added according to the instructions of the kit and the absorbance at OD 400 nm was measured. Enzyme activity was calculated according to the formula in the instructions.

### 5.15 Cellular Glucose Uptake Assay

Glucose Uptake Cell‐Based Assay Kit was purchased from Cayman Chemical. BV2 cells were plated in a 96‐well plate and incubated with 100 mM 2‐NBD‐Glucose for 20 min. The fluorescence intensity was quantified by a Cytation 5 Cell Imaging Multi‐Mode Reader (BioTek) with Ex/Em = 465/540 nm.

### 5.16 Transmission Electron Microscopy

BV2 cells were treated with DMEM containing 1 mM OA for 12 h. BV2 cells were collected after PBS cleaning and digestion with 0.025% trypsin. BV2 cells were fixed with 2.5% glutaraldehyde fixative (Adamas‐beta; F8017) for 48 h. BV2 cells were then dehydrated with graded ethanol and propylene oxide and embedded in epoxy resin. BV2 cells were cut into 70 nm sections and stained with lead citrate. BV2 cell sections were observed with a JEM‐1400 electron microscope (JEOL Ltd., Akishima, Tokyo, Japan).

### 5.17 Metabolic Measurements

BV2 cells were slant‐inserted into XF96 cell culture microplates (Agilent Technologies; 102416) and cultured overnight. The lower layer of the XF96 Extracellular Flux Assay Kit (Agilent Technologies; Q29216) was added with hydration solution overnight in a CO_2_‐free incubator at 37°C. On the second day, BV2 cells were washed with XF base Medium (Agilent Technologies; 102353) twice and cultured with XF base Medium at 37°C without CO_2_ for 1 h. Before the glycolysis assay, glucose, OLI, and 2‐deoxy‐d‐glucose were diluted with XF base medium. OLI, FCCP, and rotenone/antimycin A were diluted with XF base medium before FAO and mitochondrial respiratory capacity measurements. After adding the diluted drug to the upper layer of the XF96 Extracellular Flux Assay Kit, Seahorse XF Pro Analyzer (Agilent Technologies) was used for detection. The lower layer was replaced with CO_2_‐free BV2 cells cultured for 1 h and tested again with a Seahorse XF Pro Analyzer.

### 5.18 Flow Cytometry

BV2 cells were washed with PBS and digested with 0.025% trypsin. Then, BV2 cells were collected and incubated with antibodies for 30 min or 1 µg mL^−1^ BODIPY 493/503 for 5 min. The cells were washed with PBS and centrifuged. Then, the cells were suspended with PBS and detected by a flow cytometer (MoFlo XDP; Beckman Coulter); antibodies: anti‐CD11B APC (Invitrogen; 17‐0112‐82) and anti‐CD45 FITC (Invitrogen; 11‐0451‐81).

### 5.19 Open Field Experiment

The open field experiment was performed in a 40 × 40 cm^2^ box (RWD Life Science Co., Ltd.). The mice were habituated to the environment the day before the experiment. The mouse was placed in the central location of the box and the video tracking system started to record movement for every 10 min. The distance and time traveled in the total zone and the center zone was calculated. The video tracking system was Smart 3.0 by Panlab.

### 5.20 Y Maze Experiment

In the training phase, the mouse is placed in the Y maze with two open arms and one blocked (the novel arm) for 10 min to explore. After 3 h, the test phase begins. The novel arm is unblocked, and the mouse is reintroduced to the maze to explore all three arms for 10 min.

### 5.21 Morris Water Maze Experiment

A hidden platform was submerged just below the water surface in one of the quadrants. Mice underwent training for 7 consecutive days, with four trials per day, to find the hidden platform using spatial cues. On day 8, a probe trial was conducted with the platform removed to assess memory retention.

### 5.22 Statistical Analysis

The visualization of lipidomics analysis results is implemented by prcomp, ggplot2, and complexHeatmap packages in R (Version 4.0.2). Correlation scores for ATP11B and KEGG enriched pathways were calculated using the AUCell package of R. Immunofluorescence and immunohistochemistry measurement was performed by ImageJ 1.8.0. The fluorescence intensity of BODIPY 558/568 C12 and the number of BODIPY+ LDs was calculated by ImageJ 1.8.0. GraphPad Prism 8 software was used for all statistical analysis. All data are expressed as the mean ± SEM and analyzed by two‐tailed Student's *t*‐test or one‐way ANOVA. *p < 0.05, **p < 0.01, ***p < 0.001, ****p < 0.0001.

## Author Contributions

J. W., C. Z., and X. D. conceptualized and supervised experiments. Y. Z. and S. Z. designed and performed experiments, analyzed data, and wrote and edited the manuscript. X. Z. and P. W. analyzed data and wrote and edited the manuscript. Y. Y., L. W., Z. C., and J. Z. wrote and edited the manuscript. Y. C. performed lipid UPLC‐QE MS and analyzed data. All authors have read and approved the final manuscript.

## Conflicts of Interest

All authors declare no conflicts of interest.

## Ethics Statement

The study was approved by the Animal Ethics Committee of Shanghai University (Approval No. ECSHU 2023–028, Date: June 13, 2023).

## Supporting information



Supporting Information

## Data Availability

All data needed to evaluate the conclusions in the paper are present in the paper or the Supporting Information. The data that support the findings of this study are available from the corresponding author upon reasonable request.
