# Supplementary material for: ATP11B Modulates Microglial Lipid Metabolism and Alleviates Alzheimer's Disease Pathology
Source: MedComm (2020). 2025 Mar 22;6(4):e70139. doi: 10.1002/mco2.70139 (PMC11928880; doi:10.1002/mco2.70139)
Supplement: Supplementary file 1 — Supporting Information [file MCO2-6-e70139-s001.pdf]

Title: ATP11B modulates microglial lipid metabolism and alleviates Alzheimer's disease pathology

Author names and affiliations: Yuchen Zhang[1,2], Shibo Zhang[1], Xuyu Zhao[1], Peiru Wu[1], Yiwei Ying[1,2], Lingling Wu[1,2], Junyi Zhuang[1], Zixin Chen[1], Yufan Chao[2], Xin Dong[2,*], Robert Chunhua Zhao[1,3,4,5,*], Jiao Wang[1,*]

1 School of Life Sciences, Shanghai University, Shanghai 200444, China

2 School of Medicine, Shanghai University, Shanghai 200444, China

3 Institute of Basic Medical Sciences Chinese Academy of Medical Sciences, School of Basic Medicine Peking Union Medical College, Beijing, China.

4 Centre of Excellence in Tissue Engineering, Chinese Academy of Medical Sciences, Beijing, China.

5 Beijing Key Laboratory of New Drug Development and Clinical Trial of Stem Cell Therapy (BZ0381), Beijing, China.

Corresponding author:

Jiao Wang, School of Life Sciences, Shanghai University, 99 Shangda Road, 200444, Shanghai, China.

E-mail: jo717@shu.edu.cn
Phone number: +86-021-66132512

Robert Chunhua Zhao, School of Life Sciences, Shanghai University, 99 Shangda Road, 200444, Shanghai, China.

Xin Dong, School of Medicine, Shanghai University, 99 Shangda Road, 200444, Shanghai, China.

Present/permanent address: Shanghai University, 99 Shangda Road, 200444, Shanghai, China.

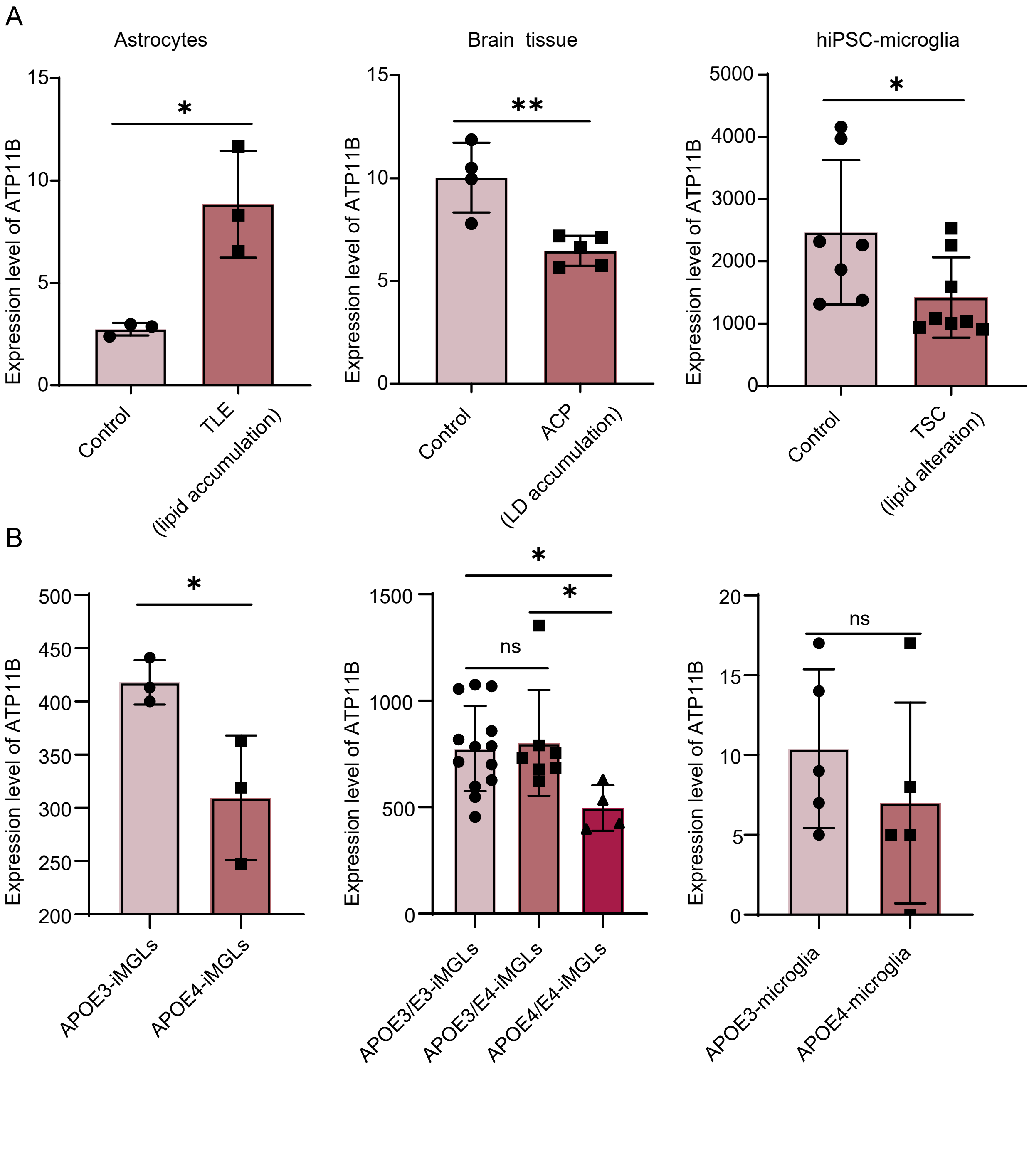
A
Astrocytes
Expression level of ATP11B
Control
TLE
(lipid accumulation)
Brain tissue
ACP
(LD accumulation)
hiPSC-microglia
TSC
(lipid alteration)
B
APOE3-iMGLs
APOE4-iMGLs
APOE3/E3-iMGLs
APOE3/E4-iMGLs
APOE4/E4-iMGLs
ns
APOE3-microglia
APOE4-microglia

Figure S1 The Expression of *Atp11b* based on Gene Expression Omnibus data. A. The Expression of *Atp11b* in human samples of diseases with abnormal lipid metabolism in the nervous system based on Gene Expression Omnibus data. Two-tailed Student's t test. B. The Expression of *Atp11b* in iMGLs and SV40 cells of ApoE ε4 allele based on Gene Expression Omnibus data. Two-tailed Student's t test.

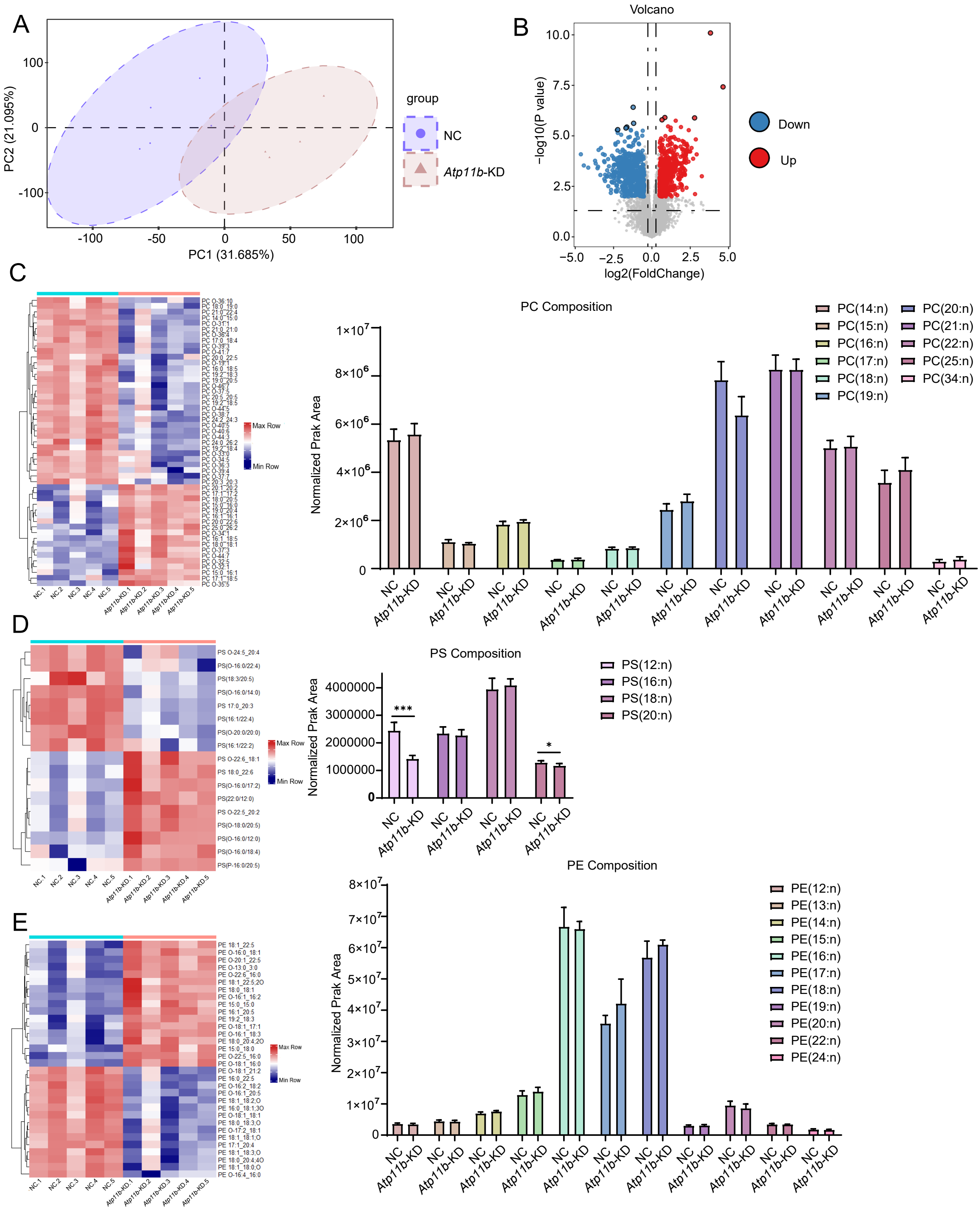
A
PC2 (21.095%)
PC1 (31.685%)
group
NC
Atp11b-KD
B
Volcano
-log10(P value)
log2(FoldChange)
Down
Up
C
Max Row
Min Row
PC Composition
Normalized Prak Area
PC(14:n)
PC(15:n)
PC(16:n)
PC(17:n)
PC(18:n)
PC(19:n)
PC(20:n)
PC(21:n)
PC(22:n)
PC(25:n)
PC(34:n)
D
PS Composition
PS(12:n)
PS(16:n)
PS(18:n)
PS(20:n)
***
*
E
PE Composition
PE(12:n)
PE(13:n)
PE(14:n)
PE(15:n)
PE(16:n)
PE(17:n)
PE(18:n)
PE(19:n)
PE(20:n)
PE(22:n)
PE(24:n)

Figure S2 The PCA and phosphoric acid composition of BV2 cells lipidomics. A. PCA plot of the profile of *Atp11b*-KD relative to NC BV2 cells. B. Volcano plot showing differentially expressed lipids in *Atp11b*-KD versus NC BV2 cells. C-E. Heat map and composition of PCs (C), PSs (D) and PEs (E) that were found dysregulated between NC and *Atp11b*-KD BV2 cells. Two-tailed Student's t test (n = 5 of two experiments).

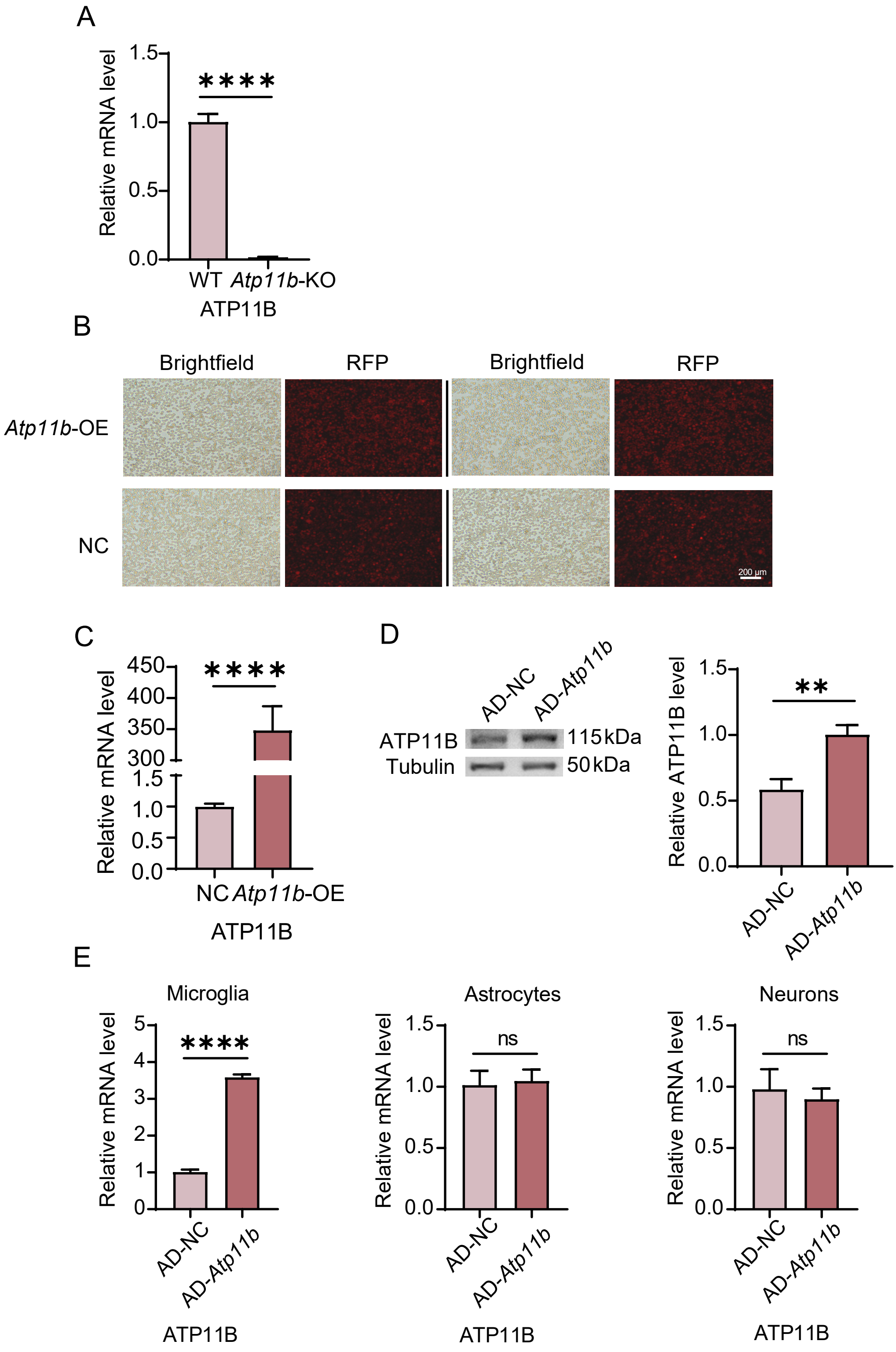
A
Relative mRNA level
1.5
1.0
0.5
0.0
****
WT
Atp11b-KO
ATP11B
B
Brightfield
RFP
Brightfield
RFP
Atp11b-OE
NC
200 μm
C
Relative mRNA level
450
400
350
300
1.5
1.0
0.5
0.0
****
NC
Atp11b-OE
ATP11B
D
AD-NC
AD-Atp11b
ATP11B
Tubulin
115kDa
50kDa
Relative ATP11B level
**
AD-NC
AD-Atp11b
E
Microglia
Astrocytes
Neurons
Relative mRNA level
****
ns
ns
5
4
3
2
1
0
AD-NC
AD-Atp11b
ATP11B
ATP11B
ATP11B

Figure S3 Validation of acutely isolated microglia and lentivirus -transfected microglia. A. qPCR measurement of mRNA levels of *Atp11b* in microglia acutely sorted from WT and *Atp11b*-KO mice. Two-tailed Student's t test (n = 4 of two experiments). B. Bright field and fluorescence images of NC and *Atp11b*-OE BV2 cells. C. qPCR measurement of mRNA levels of *Atp11b* in NC and *Atp11b*-OE BV2 cells. Two-tailed Student's t test (n = 4 of two experiments). D. Western blot images and quantification of ATP11B level in NC and *Atp11b*-OE BV2 cells both treated with Aβ for 24h. Two-tailed Student's t test (n = 3 of two experiments). E. qPCR measurement of mRNA levels of *Atp11b* in microglia, astrocytes, and neurons acutely sorted from AD-NC and AD-*Atp11b* mice. Two-tailed Student's t test (n = 4 of two experiments).
